# Gallotannin Imposes S Phase Arrest in Breast Cancer Cells and Suppresses the Growth of Triple-Negative Tumors *In Vivo*

**DOI:** 10.1371/journal.pone.0092853

**Published:** 2014-03-21

**Authors:** Tiejun Zhao, Qiang Sun, Sonia V. del Rincon, Amanda Lovato, Maud Marques, Michael Witcher

**Affiliations:** The Lady Davis Institute and Segal Cancer Center of the Jewish General Hospital, McGill University, Montreal, Quebec, Canada; Weizmann Institute of Science, Israel

## Abstract

Triple-negative breast cancers are associated with poor clinical outcomes and new therapeutic strategies are clearly needed. Gallotannin (Gltn) has been previously demonstrated to have potent anti-tumor properties against cholangiocarcinoma in mice, but little is known regarding its capacity to suppress tumor outgrowth in breast cancer models. We tested Gltn for potential growth inhibitory properties against a variety of breast cancer cell lines *in vitro*. In particular, triple-negative breast cancer cells display higher levels of sensitivity to Gltn. The loss of proliferative capacity in Gltn exposed cells is associated with slowed cell cycle progression and S phase arrest, dependent on Chk2 phosphorylation and further characterized by changes to proliferation related genes, such as cyclin D1 (CcnD1) as determined by Nanostring technology. Importantly, Gltn administered orally or via intraperitoneal (IP) injections greatly reduced tumor outgrowth of triple-negative breast cells from mammary fat pads without signs of toxicity. In conclusion, these data strongly suggest that Gltn represents a novel approach to treat triple-negative breast carcinomas.

## Introduction

Breast cancer is an epidemic afflicting 11–12% of North American women [1]. Clinically, breast cancer is generally stratified into four major subtypes; luminal A, luminal B, Her2+ and triple-negative. Patients with triple-negative tumors are at highest risk for recurrence and have the shortest overall survival [2]. Triple-negative patients are unresponsive to trastuzumab, or endocrine-targeting therapies due to the absence of Her2 and Estrogen receptor targets. Currently, triple-negative tumors are generally targeted with surgery and traditional cytotoxic agents such as paclitaxel. However, conventional chemotherapies like paclitaxel or cyclophosphamide can have severe side effects and may actually promote tumor progression in some cases [3], [4], [5]. Clearly, new therapeutic strategies are needed to combat triple-negative breast cancer both at time of onset, and if necessary, at recurrence.

Many studies have drawn associations between the consumption of products high in polyphenols (such as green tea) and reduced incidence of cancer, including breast cancer [6], [7]. Pharmacologic application of several polyphenols such as resveratrol, have been shown to have moderate growth inhibitory effects on breast cancer growth in xenograft models [8], [9]. Gltn is a naturally occurring polyphenol derived from diverse sources. Supporting epidemiological data relating to polyphenol intake, dietary Gltn works as a prophylactic against tumor initiation. Dietary Gltn reduced the frequency and number of both stomach and lung tumors in a carcinogen-induced mouse model of cancer [10]. Further studies have reported Gltn to have potent growth inhibitory properties against xenograft models of choliangiocarcinoma and colon cancer [11], [12]. Importantly, neither of these reports showed Gltn to generate discernible off-target toxicity *in vivo*. Against breast cancer, Gltn has been shown to reduce the growth of Brca2 mutated cells *in vitro* through induction of double strand breaks, similar to what is observed with Parp inhibitors [13]. The mechanism through which Gltn suppresses proliferation of other tumor types remains unclear. Also unclear, is its capacity to work against breast cancer cells with wild type Brca *in vitro* or *in vivo*.

We now show that Gltn exhibits strong growth inhibitory properties against a panel of breast cancer lines, with triple-negative cells showing greater sensitivity than luminal cells. We demonstrate for the first time that Gltn-mediated growth inhibition is characterized by cell cycle arrest in S phase, dependent on activation of Chk1. Importantly, using a preclinical xenograft model, we also find the outgrowth of triple-negative breast tumors is considerably repressed by low dosage Gltn *in vivo*.

## Materials and Methods

### Cell culture reagents

MDA-MB-468, BT-20, HCC1937, MDA-MB-435, MDA-MB-436, T47D, Sum-149 and BT-474 were grown as recommended by ATCC. Gltn was purchased from Sigma. Gltn was prepared fresh for each drug treatment in PBS. Control plates were exposed to PBS as a vehicle control. Gltn was repurchased regularly to maintain stocks that were not oxidized through exposure to air. For cell viability assays, 1×10^4^ cells were initially seeded in 24 or 12 well plates. Gltn was added fresh daily and cells were counted using a hemocytometer and trypan blue exclusion. Viability assays were carried out in triplicate and repeated at least three times. Chk2 inhibitor (EMD Millipore, Cat# 220486) was added to a final concentration of 75 nM simultaneously with Gltn.

### Western Blotting, Immunostaining and antibodies

Whole cell lysates were separated using standard SDS-Page electrophoresis techniques, followed by transfer to BioRad nitrocellulose membranes and probing with the antibodies listed below. For Immmunohistochemistry (IHC), tumors were excised 30 days after drug treatment began and whole tumors were fixed in formalin. IHC was carried out by the Molecular Pathology core facility at the Jewish General Hospital using standardized procedures with the Ventana Discovery automated IHC machine. Antibodies were used at a dilution of 1:50. Immunofluoresence (IF) for γH2A.X was done as previously described [14]. Doxorubicin-exposure served as a positive control for γH2A.X induction. For IF, γH2A.X was used at a dilution of 1∶200 and allowed to bind cells at room temperature for two hours before washing. The antibodies used as probes for Western and IHC were as follows; Anti-phospho-H2A.X (EMD Millipore), CcnD1, CcnE1, laminB, βTubulin (Santa Cruz), Chk1, Chk2, phospho-Chk1 and phospho-Chk2 (Thr68) (Cell signaling), Cdc25a, phosphor-Cdc25a (Abnova), β-Actin (Sigma) and CcnA1 (BD biosciences).

### Cell cycle analysis

Annexin V labeling and propidium iodide staining was carried out according to the recommendations of the manufacturer (BD biosciences). Quantification and analyses were carried out using Flow assisted cytometry and FlowJo software. For cell synchronization, cells were blocked initially with thymidine (2 mM, 24 hrs) followed by Nocodozole (100 ng/ml, 12 hrs) to achieve G2/M arrest. Cells released from cell cycle arrest were subsequently analyzed for cell cycle progression via propidium iodide and BrdU staining. All BrdU reagents including BrdU, 7-AAD and FITC-coupled anti-BrdU antibodies were purchased from BD biosciences (Cat# 557891). For BrdU labeling, BrdU was added to cells for the indicated time prior to harvest, after which cells were processed as recommended by manufuacturer. BrdU labeling data was analyzed using FlowJo software. All experiments were repeated minimally three times.

### NanoString and qRT-PCR analyses of gene expression

For NanoString, a total of 100 ng RNA was analyzed using the NanoString nCounter Analysis System at the University Health Network Microarray Centre,Toronto, Canada. RNA was isolated using total RNA prep kit from Qiagen. Gltn was added daily for the indicated time periods. Nanostring results reflect the mean of three independent drug treatments. The nCounter human cancer reference code set was employed to probe 230 cancer-related genes simultaneously. Background counts indicating negative expression was set at two standard deviations above the average of internal negative controls. Genes having no expression in the cell lines examined were excluded from heat maps. Raw counts were normalized using the mean of four internal positive controls. The normalized counts were then compared to untreated control cells to determine fold change in mRNA levels. Gene expression was validated using qPCR with cDNA produced using the Quanta reverse transcriptase kit. RNA used in validation experiments was acquired from experiments independent of those used to isolate RNA for Nanostring analysis.

### Xenograft assays

All animal experiments were carried out in accordance with approved standard operating procedures and ethical standards of the Lady Davis Institute and McGill University, Animal Use Protocol # 2011-7003. The animal studies included herein were specifically approved by our ethics board. One million MDA-MB-468 cells in PBS were injected into the fat pad of the first mammary gland of nu/nu athymic mice (Charles River laboratories). Tumors were allowed to develop until palpable (∼ 0.7 – 1 mm in diameter). After tumor outgrowth was detected, mice were exposed to 0.5% Gltn in drinking water or daily IP injections of 10 mg/kg Gltn in PBS or PBS as a control. Drinking water +/- Gltn was replaced daily and tumor growth followed over a 30 day period. Tumor size was measured using digital calipers and volume determined using the formula V  =  ½ × length (mm) × width (mm)^2^. Patient-ready paclitaxel was purchased from the in-house pharmacy at the Jewish General Hospital (Montreal). Paclitaxel was injected twice weekly at indicated concentrations.

### Statistical analysis

To calculate experimental p values, a two-sided Student t test was applied for comparison of continuous variables between two groups. Differences were considered significant when the p values were<0.05. IC50 was determined using Graph Pad Prism nonlinear regression, Sigmoidal curve best fit model. For Ingenuity pathway prediction, p-values were assigned by the software based on Fisher exact test scores, dependent upon the number of genes that mapped to a particular biological pathway.

## Results

### Gltn inhibits breast cancer cell proliferation through cell cycle arrest

**Figure 1:**
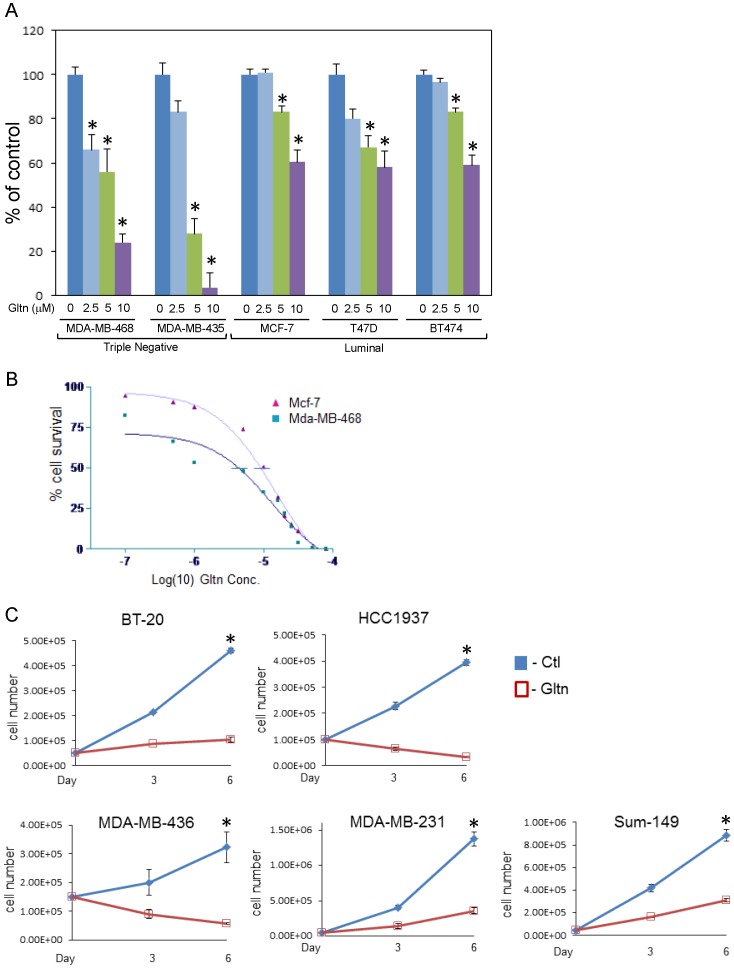
Gltn impairs the proliferation of triple-negative breast cancer cells *in vitro*. (**A**) Triple-negative and luminal breast cancer cells were grown with Gltn or PBS as a vehicle control for 5 days at the indicated dose. Cell numbers were determined using trypan blue exclusion and hemacytometer in n = />3 experiments +/- S.E.* denotes significant differences compared to controls with p values  = /< 0.05 (**B**) Sigmoidal dose response curves comparing Gltn sensitivity in luminal MCF-7 cells with triple-negative MDA-MB-468 cells. Horizontal bars on curves represent points of IC50. (**C**) Triple-negative cell lines were seeded and exposed to 10 μM Gltn for the indicated time periods. Cells were counted as described in A. Graphs represent n = />3 experiments carried out in triplicate triplicate +/- S.E. By six days post-treatment, all cell lines showed significant differences from control group. p  = /< 0.05 as denoted by asterisks.

We tested the capacity of Gltn to reduce growth in breast cancer cells of luminal and triple-negative origin [15]. Dose curve experiments indicated that triple-negative breast cancer cells (MDA-MB-468 and MDA-MB-435) display enhanced sensitivity to Gltn exposure relative to cells with luminal characteristics (Fig. 1a,b) This was exemplified by the IC50 value of 4.99×10^−6^ μM for the triple negative line MDA-MB-468 and 11×10^−6^ for the luminal cell line MCF-7 (Fig. 1b). We extended these results and found that Gltn significantly impaired the proliferation of a panel of triple-negative cells including BT-20, Hcc-1937, MDA-MB-436, MDA-MB-231 and Sum-149 (Fig. 1c).

**Figure 2:**
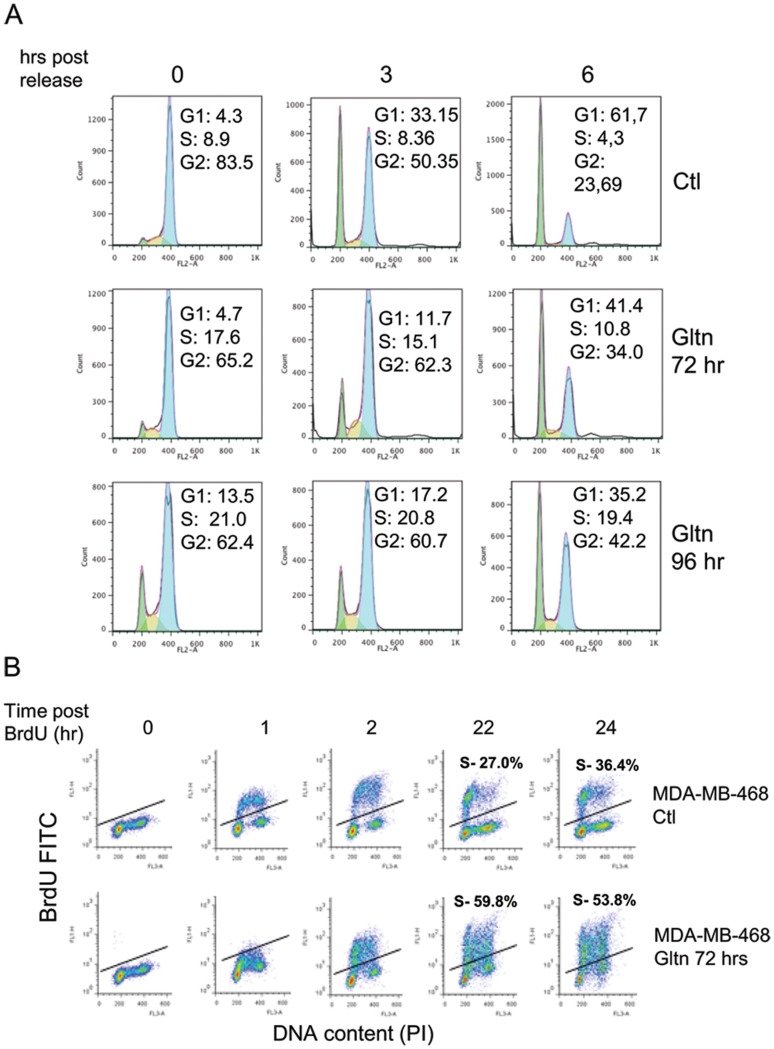
Gltn exposure results in S phase arrest. (**A**) MDA-MB-468 cells treated with Gltn for indicated times were synchronized in G2/M using double Thymidine/Nocodozole mediated arrest. Cell cycle progression was assessed after release using propidium iodide staining detected by fluorescence activated cell sorting (FACS). Results are representative of three independent experiments. (**B**) MDA-MB-468 cells were exposed to Gltn or vehicle for 72 hours prior to labeling with BrdU. Cells were collected at the indicated time periods post-BrdU labeling. BrdU incorporation was quantified using fluorescence activated cell sorting. Cells residing above the solid line are considered to reside in the S phase compartment. Results are representative of triplicate experiments.

To gain insight into the mechanism whereby Gltn treatment inhibits cell growth, we first carried out propidium iodide and annexin V staining to test for cell cycle defects or apoptosis in response to Gltn exposure. At 72 hr post treatment we observed slowed growth (Fig. 1) in the absence of cell death (Fig. S1). This was consistent with observations of cell morphology (Fig. S1b). Next, we investigated the possibility that Gltn alters cell cycle. We utilized thymidine and nocodoczole to synchronize cells in G2 phase and followed the cell cycle progression after release. These experiments show Gltn treated cells resist progression through S phase during the synchronization procedure (Fig. 2a). Upon release from cell cycle blockage, Gltn exposed cells cycle more slowly through G2 than their untreated counterparts and accumulate in S phase (Fig. 2a). These experiments suggest Gltn was imposing an S phase arrest on treated cells. To more accurately quantify cells residing in S phase, we labeled asynchronous MDA-MB-468 control and Gltn treated cells with BrdU. Cells exposed to Gltn for 96 hours showed a stark accumulation of S phase cells as measured by BrdU incorporation (Fig. 2b). By 22 hours post-BrdU labeling, Gltn exposed MDA-MB-468 cells were over two fold more likely to be found in S phase than control cells. Together, these data clearly indicate that Gltn impairs cell cycle progression leading to S phase arrest. Even though MDA-MB-435 cells were more sensitive to growth inhibition by Gltn than other cell lines tested, they also exhibited accumulation in S phase (Fig. S1c). However, MDA-MB-435 cells progressed to an apoptotic phase more readily than other triple negative cells (data not shown).

**Figure 3:**
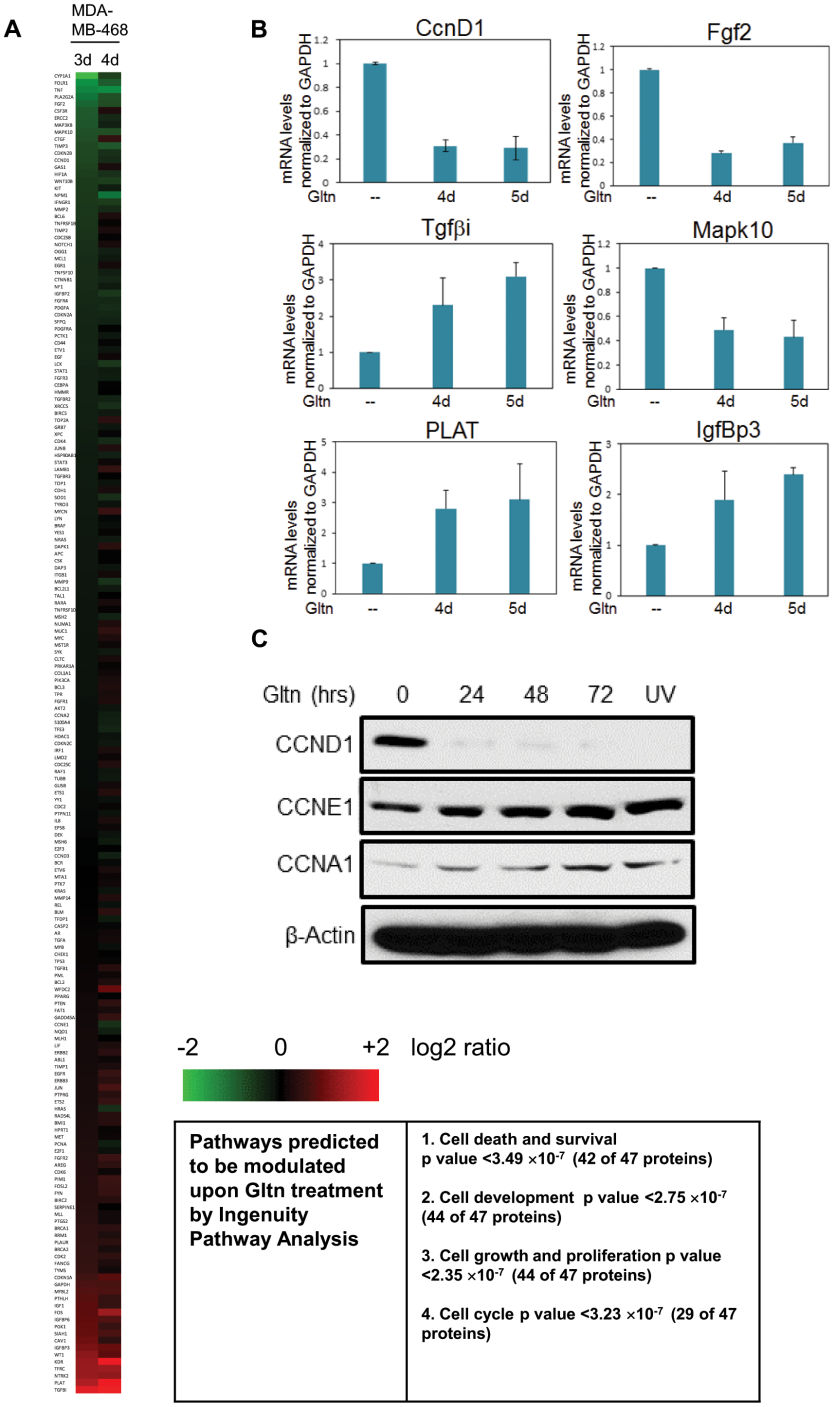
Gltn regulates the expression of proliferation related genes. (**A**) MDA-MB-468 cells were treated with Gltn in triplicate for three or four days as indicated. RNA from treated cells or controls were isolated and gene expression analyzed by Nanostring technology. The results represent the mean fold-change of three independent drug treatments. (**B**) qPCR validation of genes regulated in MDA-MB-468 cells by Gltn. n = />2 experiments carried out in triplicate +/- S.D. (**C**) Western blotting of cyclin expression after Gltn treatment for the indicated time periods. UV light serves as a positive control for CcnD1 degradation.

We utilized Nanostring technology to identify a gene expression signature associated with Gltn induced growth arrest. Gene expression profiles were analyzed from cells treated with Gltn for 3 or 4 days (Fig. 3a, full data set shown as Table S1) and validated by qPCR (Fig. 3b). Interestingly, less than 15% of the 230 genes examined showed significant changes after Gltn treatment (two-fold or greater), indicating a specific pathway or network is being targeted by Gltn. We analyzed the function of those genes whose levels changed by 2-fold or greater upon Gltn treatment using Ingenuity Pathway Analysis software. p-values were assigned by the software based on Fisher exact test scores, dependent upon the number of genes that mapped to a particular biological pathway. Consistent with our growth assays, computational analysis predicted that 27 of the 31 Gltn-regulated genes would target cell growth and proliferation (p value < 1.12×10^−4^). These include downregulation of the growth factor Fgf2 and increased expression of the tumor suppressor TgfβI [16], [17]. Notably, Nanostring and qPCR analysis highlighted that CcnD1 mRNA levels were significantly decreased upon Gltn treatment (Fig. 3a, b and Fig. S2). Cyclins play a pivotal role in cell cycle progression and several of these act as oncogenes. Therefore, the suppression of CcnD1 potentially represents an important biomarker predicting responsiveness to Gltn. We expanded upon our Nanostring and qPCR data to probe for protein expression of several key oncogenic cyclins, including CcnD1, CcnE1 and CcnA1 in Gltn treated cells. Of these, CcnD1 was exclusively downregulated, showing specificity of the pathways influenced by Gltn (Fig. 3c). This is consistent with other studies that have described a loss of CcnD1 upon S phase arrest in response to the polyphenol resveratrol [18]. However, in contrast to this study which saw CcnD1 levels reduced after only two hours of treatment, we saw no reduction of CcnD1 levels at these early time periods in either MDA-MB-468 or MDA-MB-435 cells (Fig. S2).

### Gltn mediated S phase arrest is associated with Chk2 activation

**Figure 4:**
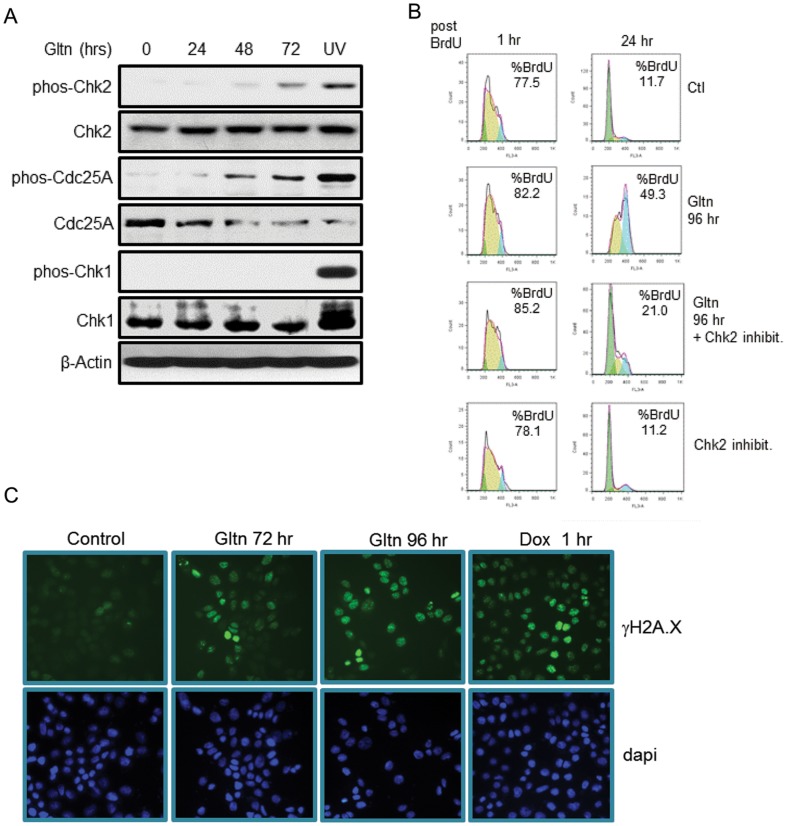
Chk2 is activated in response to Gltn. (**A**) Western blotting of Check point kinases in response to Gltn. UV light serves as a positive control for Chk1 and Chk2 phospohorylation. Results are representative of triplicate experiments. (**B**) MDA-MB-468 cells were treated with Gltn for 72 hours prior to BrdU labeling. BrdU was quantified using FACs and results are representative of three independent experiments. (**C**) Immunofluorescent images of γH2A.X in Gltn treated cells. Cells were treated with Gltn daily for the indicated time periods and probed with an anti-Ser-129-H2A.X antibody for two hours. Doxorubicin treatment serves as a positive control for DNA damage. Results are representative of three independent experiments.

Knockdown studies show that loss of CcnD1 generally results in G1 arrest [19]. Hence, even though loss of CcnD1 could contribute to the growth arrest observed in Gltn treated cells, it alone is unlikely to account for the S phase arrest observed in these cells. A previous study observed that the growth inhibition of colon cancer cells exposed to Gltn was associated with impaired NF-κβ activity [12]. In breast cancer cells, Gltn as a single agent does not impair NF-κβ activity, and actually a slight increase in NF-κβ binding was observed by gel mobility shift analysis (Fig. S3). However, Gltn can block TNF-mediated activation of NF-κβ as previously described (Fig. S3) [12]. The lack of impediment to the NF-κβ pathway is not surprising when one considers that inhibition of NF-κβ signaling has not been reported to induce S phase arrest [20], [21]. Therefore, we investigated the potential impact of Gltn on checkpoint kinase activation. Chk1 and Chk2 activation are key mediators of S to G2 phase progression and activation of Chk2 in particular controls an intra-S phase checkpoint resulting in cell cycle arrest within S phase [22]. We see that Gltn specifically stimulates a dramatic increase of Chk2 phosphorylation, but not of Chk1, (Fig. 4a). This activation was apparent in both MDA-MB-468 and MDA-MB-435 cells within eight hours post-treatment (Fig. S4). Chk2 activation targets CDC25A for phosphorylation and subsequent degradation [23]. In keeping with Chk2 activation by Gltn, we also observed Cdc25A phosphorylation accompanied by lowered expression in response to Gltn (Fig. 4a). To verify that the accumulation of cells in S phase after Gltn exposure was due to Chk2 activation we utilized an inhibitor of Chk2 [24], [25]. Dramatically, Chk2 inhibition reduced the forced accumulation of cells in S phase by Gltn by over 28% (Fig. 4b). These data show, for the first time, that growth suppression in response to Gltn is associated with S phase arrest mediated by Chk2 activation.

Chk2 is classically activated in response to DNA damage, especially ionizing radiation [26]. DNA damage induced Chk2 phosphorylation is observed upon exposure to numerous chemotherapeutics including the polyphenol resveratrol [27], [28]. To investigate the potential of Gltn to likewise induce DNA damage, we probed Gltn treated cells for nuclear enrichment of γH2A.X. Phosphorylation on serine 139 of the histone variant H2A.X (known as γH2A.X) accumulates in the nucleus after DNA damage and is required for repair of damaged chromatin. Untreated cells showed little γH2A.X staining by IF (Fig. 4c). However, significant levels of γH2A.X were visible at 72 hours post-treatment and by 96 hours, most cells were staining γH2A.X positive. Thus, we conclude that Gltn imposes S phase arrest on breast cancer cells through DNA damage which subsequently activates Chk2.

### Gltn effectively suppresses triple-negative tumor growth in a xenograft model

**Figure 5:**
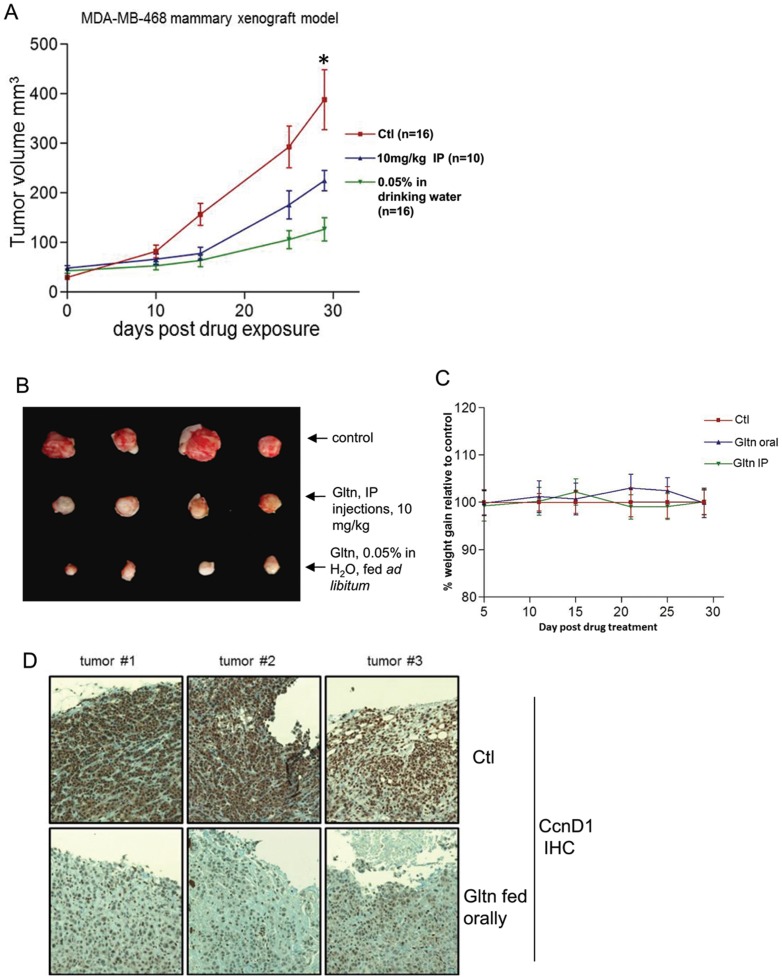
Gltn effectively suppresses triple-negative tumor outgrowth *in vivo*. (**A**) Mammary fat pads of athymic nude mice were injected with 1×10^6^ triple-negative MDA-MB-468 cells. Subsequent to tumor palpation, mice were exposed to Gltn through drinking water or intraperitoneal injections. Tumor volume was measured using digital calipers +/- S.D. The results represent two independent mouse experiments with n = /> 4 mice per group in each experiment. “*” indicates difference between experimental and control group has p<0.05. (**B**) Morphology of Gltn exposed tumors shows diminished size and increased pallor at 30 days post treatment. (**C**) Weight of mice exposed to Gltn compared to control mice +/- S.D. (**D**) Immunohistochemistry of CcnD1 levels in residual tumors from mice exposed to Gltn orally. CcnD1 expression was shown to be diminished in these tumors.

Orally administered Gltn has been shown to effectively impair cholangiocarcinoma and intraperitoneal injected Gltn has been shown to have significant effects on colon cancer outgrowth in xenograft models [11], [12]. We evaluated Gltn as a therapeutic agent against triple-negative, MDA-MB-468 outgrowth from the mammary fat pads of athymic nude mice administered either orally (0.5% in drinking water) or via intraperitoneal injection (10 mg/kg/5 injections per week). Statistically significant differences in tumor outgrowth were apparent within fifteen days of Gltn treatment (Fig. 5a). Interestingly, IP administered Gltn was less effective at sustaining growth inhibition than low dose Gltn given orally (Fig. 5a,b). Even after thirty days, tumors exposed to Gltn orally showed little growth (Fig. 5a). Gltn exposed tumors were also distinguished by their pallor, indicative of necrotic tissue [29] (Fig. 5b).Toxicity of chemotherapeutic agents is of great clinical concern. Not unexpectedly, these low doses of Gltn did not induce any signs of toxicity as determined by overall weight loss or liver damage (Fig. 5c and Fig. S5). Histological analysis of the residual tumor remaining after exposure to oral Gltn demonstrated markedly reduced CcnD1 expression (Fig. 5d) and elevated phosphorylation of Chk2 (Fig. S6). These data indicate that the reduced proliferation of tumor tissue *in vivo* is consistent with the decreased CcnD1 levels observed in response to Gltn exposure *in vitro* (Fig. 2 and 4).

Paclitaxel is a cytotoxic agent commonly used to treat triple-negative tumors. Therefore we compared the sensitivity of MDA-MB-468 xenografts to paclitaxel with Gltn. Surprisingly, paclitaxel at non-toxic concentrations (5 mg/kg) showed little impact on tumor growth (data not shown) and even paclitaxel administered at 20 mg/kg showed little efficacy, while being highly toxic (Fig. S7). Altogether, these results demonstrate that Gltn is an effective, non-toxic anti-cancer therapeutic that is superior to paclitaxel in preclinical animal models of triple-negative breast cancer.

## Discussion

The goal of this study was to determine the applicability of Gltn for the growth inhibition of breast cancer cells. Amongst a panel of breast cancer cell lines, we observed the greatest cytostatic effects of Gltn against triple-negative breast cancer cells. Mechanistically, our cell cycle analysis and BrdU staining revealed that Gltn reduces cell proliferation primarily via cell cyle arrest in S phase. The practicality of targeting S phase as a mode of action for chemotherapeutics is highlighted by the Topoisomerase I inhibitor irinotecan, which is a clinically effective anti-cancer agent [30].

To explore the mechanism underlying S phase arrest we looked at potential mediators including changes to gene expression profiles using Nanostring. Gltn exposure results in a targeted panel of gene expression changes, principally involved in proliferation. Notably, these changes included downregulation of CcnD1, Fgf2 and increased expression of the tumor suppressor TgfβI. Amongst our panel of genes, TgfβI induction showed the largest increase in response to Gltn and we suggest that TgfβI represents a key biomarker for cell sensitivity to Gltn. TgfβI knockout give rise to spontaneous tumors in murine models [16]. Interestingly, several reports have shown TgfβI expression is necessary for sensitivity to cytotoxic drugs [31], [32], [33], [34], and synergistic drug interaction correlates with its expression [35]. These effects seem to be primarily mediated through activation of integrin pathways, but mTor activation may also play a role [32], [36], [37], [38].

One of the questions arising from our work is “why are triple-negative cells more sensitive to Gltn than their luminal counterparts”? One answer is differential drug uptake, and another possibility is repression of the Fgf2 gene. The proliferation of triple-negative breast cancer cells is stimulated by Fgf2 through an autocrine loop [39]. Fgf2 expression was observed to be substantially higher in triple-negative versus luminal, thus a reduction of Fgf2 output will severely impact the growth of these cells.

CcnD1 is integrally linked to cell proliferation, both *in vitro* and *in vivo*. Reports demonstrate that CcnD1 protein is an oncogenic driver, upregulated in up to 50% of breast tumors and associates with reduced patient survival and resistance to chemotherapeutics (reviewed in [40]). Thus, reducing CcnD1 expression is a clinically relevant goal and may represent an important facet of Gltn's anti-tumor activity.

In addition to these three key genes, the levels of IgfBp3 andMapk10 were significantly modulated after exposure to Gltn, and these might also promote the anti-tumor effects of Gltn. IgfBp3 is well established as an inhibitor of breast cancer proliferation. It acts through binding insulin-like growth factors to prevent their binding to cognate receptors, but also has anti-proliferative functions beyond this axis [41]. Several reports have shown IgfBp3 expression mediates drug sensitivity [42], [43] and represents a potential mediator of the Gltn drug response. Mapk10 (also known as Jnk3) has been associated with drug resistance and its knockdown sensitizes cells to growth inhibition to at least one anti-cancer drug [44].

While CcnD1 and Fgf2 reduction, as well as TgfβI induction, may certainly contribute to the suppressed proliferation of Gltn treated cells, there is little indication in the scientific literature that manipulating these proteins result in S phase arrest. It is well established that Chk2 is a pivotal regulator of S phase progression [45]. In response to replicative stress, DNA damage, or chromatin remodeling [46] Chk2 may be activated resulting in S phase arrest. Chk1 activation results in cell cycle arrest at either S phase or G2/M [47], [48]. Therefore, activation of either of these checkpoint kinases could underlie the S phase arrest we observed. Intriguingly, only Chk2 was phosphorylated in response to Gltn exposure, while Chk1 remained inactive. Using a Chk2 inhibitor we clearly demonstrate that Gltn-imposed S phase arrest was dependent on Chk2 activity (Fig. 4) Therefore we conclude that Gltn-induced S phase arrest results from DNA damage and is phospho-Chk2 dependent. A similar Chk2-dependent S pahse arrest is seen in response to the chemotherapeutic agent Irofulven [49]. However, the precise mechanism governing whether Chk1, Chk2 or both checkpoint kinases are activated in response to anti-cancer agents remains unclear, but may be due to differential targeting of spindle assembly [50].

Gltn is “generally regarded as safe” by the FDA. Previous studies show Gltn has high bioavailability in rodents with little toxicity. In fact, signs of liver toxicity are not apparent in rats administered Gltn until doses of 150 mg/kg or greater are administered for 290 days [51]. Our data indicate that low dosage Gltn readily reaches tumor tissue in the mammary gland. In our xenograft model of triple negative breast cancer, Gltn worked as an effective therapeutic with little off-target cytotoxicity. Consistent with our *in vitro* data, CcnD1 expression was also decreased in tumors from Gltn treated animals. Based on the potential importance of CcnD1 as a clinical target, and the strong anti-tumor effects of Gltn seen in preclinical models of triple-negative breast and cholangiocarcinoma, we suggest that Gltn should be considered for clinical trials as an anti-cancer agent.

## Supporting Information

Figure S1 **Gltn does not induce apoptosis at 72 hours post-treatment.**
(TIFF)

Figure S2 **Gltn does not reduce CcnD1 protein levels as early time points.**
(TIFF)

Figure S3 **Gltn does not inhibit NF-κβ binding to consensus site.**
(TIFF)

Figure S4 **Activates Chk2 in MDA-MB-435 cells.**
(TIFF)

Figure S5 **Mice exposed to Gltn show no obvious signs of toxicity.**
(TIFF)

Figure S6 **Gltn exposed tumors display increased Chk2 phosphorylation.**
(TIFF)

Figure S7 **Paclitaxel displays high toxicity and little effecacy against MDA-MB-468 xenografts.**
(TIFF)

Table S1 **MDA-MB-468 fold change (Gltn/Ctl), shown in** **Fig.3** **.**
(XLSX)
