# Supplementary figures and images for: Gallotannin Imposes S Phase Arrest in Breast Cancer Cells and Suppresses the Growth of Triple-Negative Tumors In Vivo
Source: PLoS One. 2014 Mar 21;9(3):e92853. doi: 10.1371/journal.pone.0092853 (PMC3962455; doi:10.1371/journal.pone.0092853)

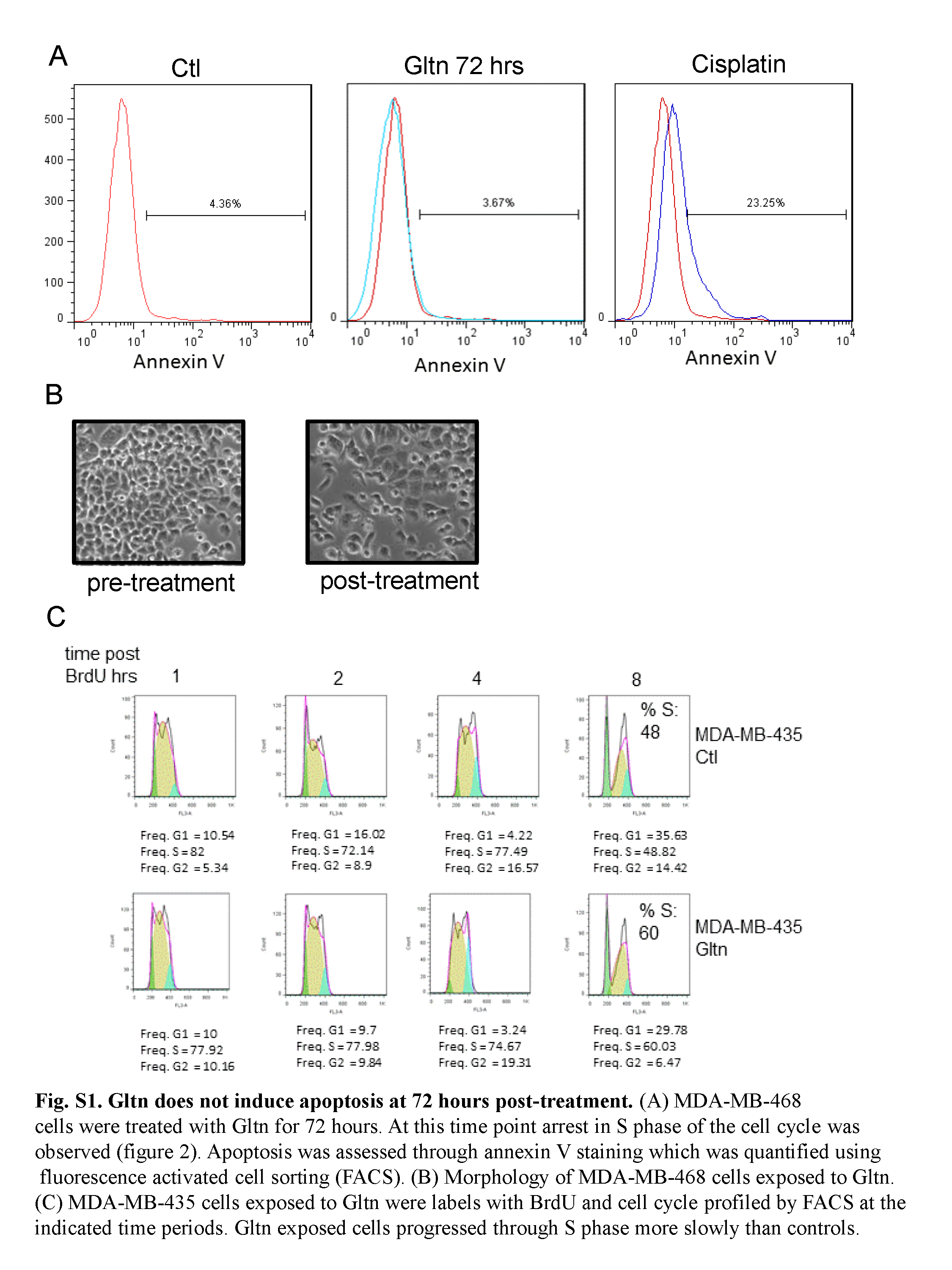

Supplement: Figure S1 — Gltn does not induce apoptosis at 72 hours post-treatment. (TIFF) [file pone.0092853.s001.tiff]

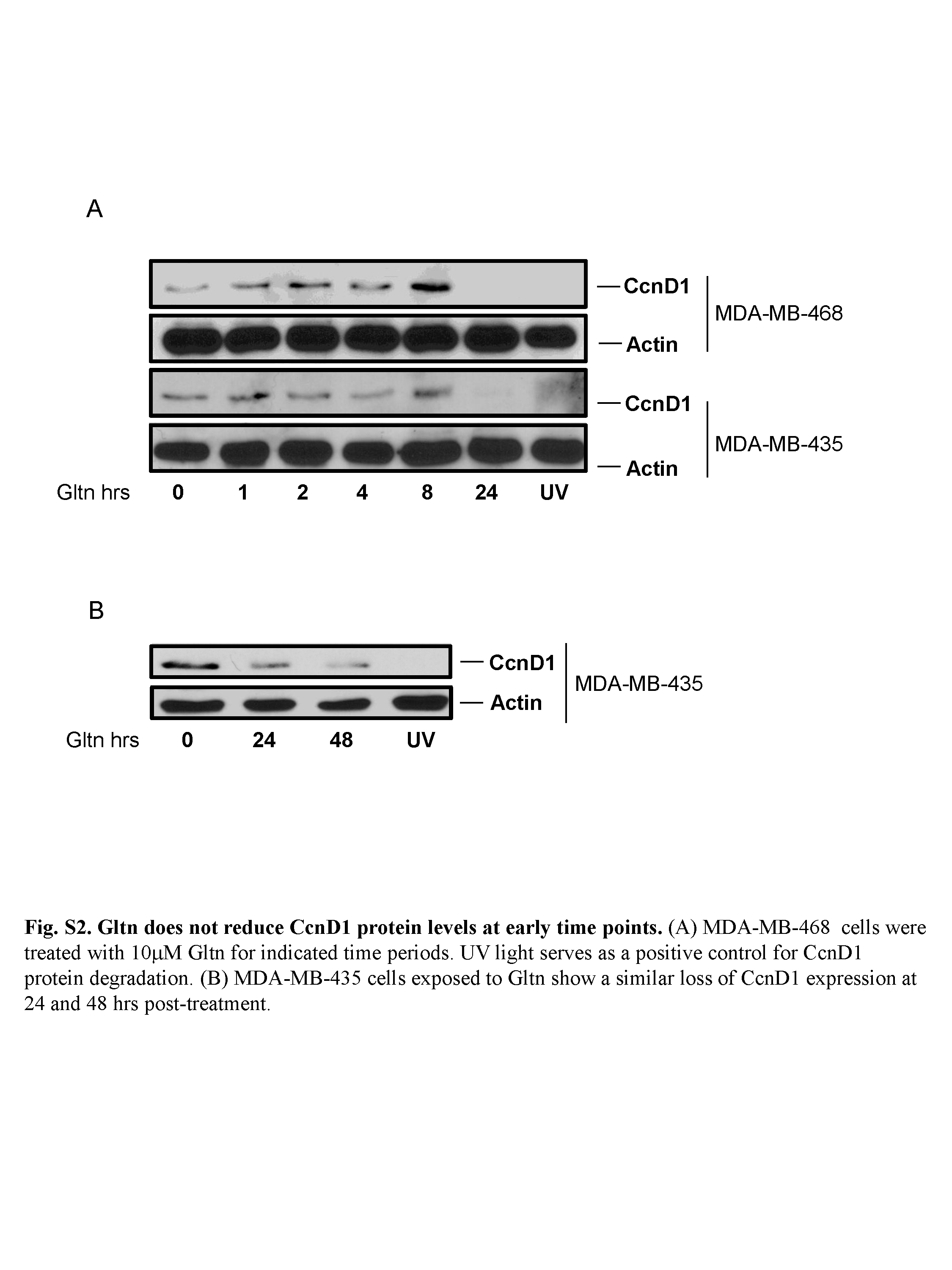

Supplement: Figure S2 — Gltn does not reduce CcnD1 protein levels as early time points. (TIFF) [file pone.0092853.s002.tiff]

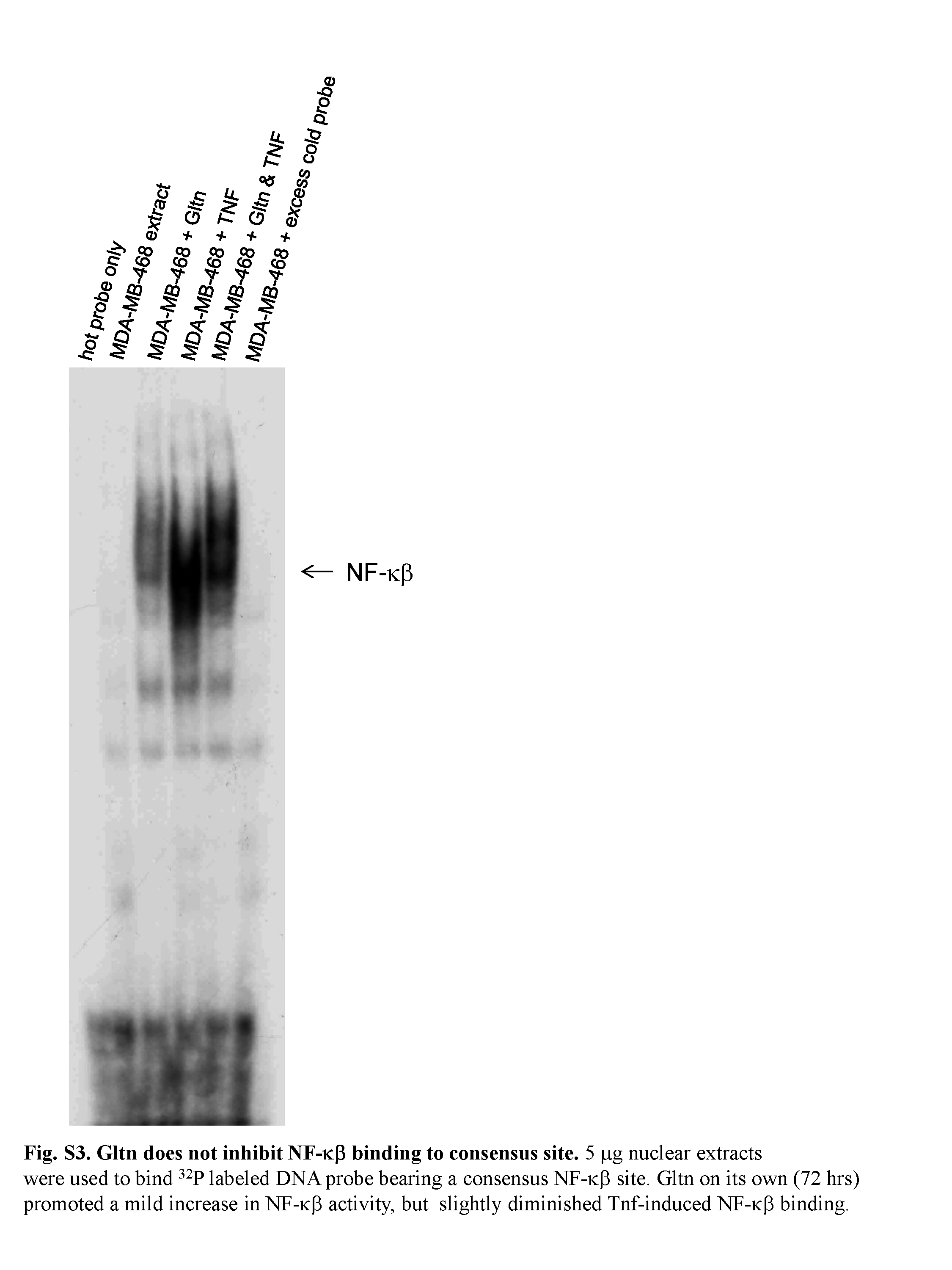

Supplement: Figure S3 — Gltn does not inhibit NF-κβ binding to consensus site. (TIFF) [file pone.0092853.s003.tiff]

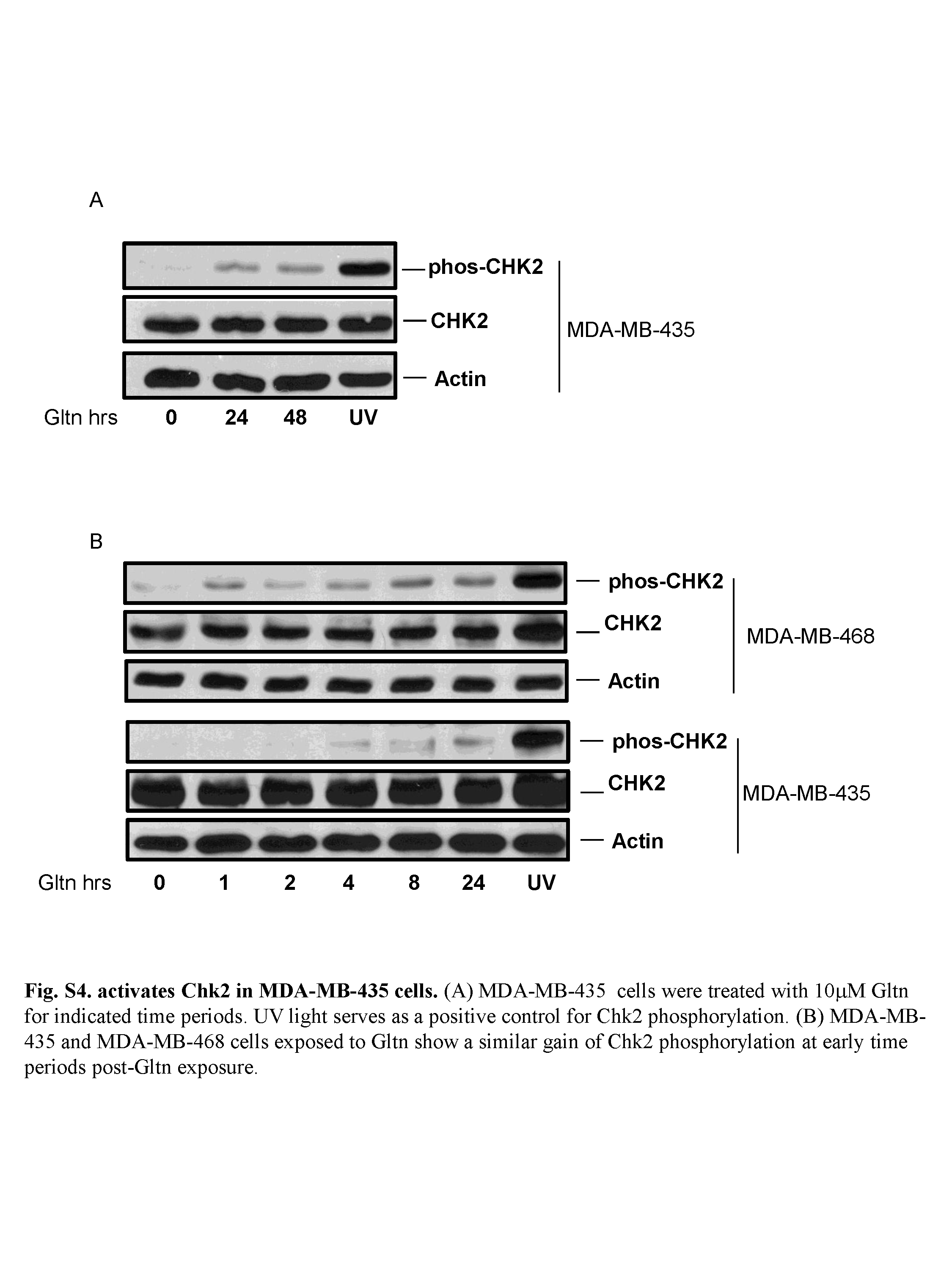

Supplement: Figure S4 — Activates Chk2 in MDA-MB-435 cells. (TIFF) [file pone.0092853.s004.tiff]

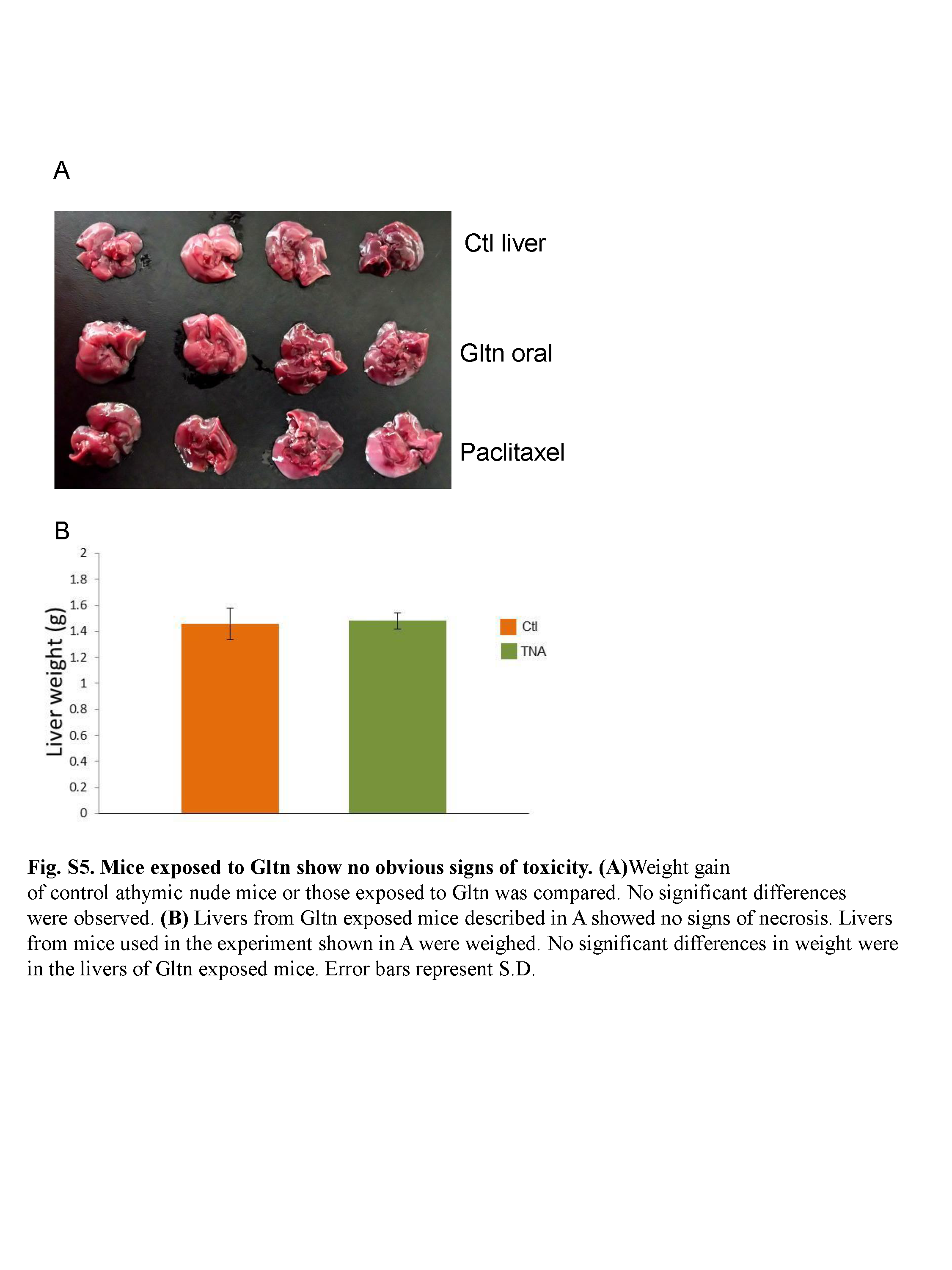

Supplement: Figure S5 — Mice exposed to Gltn show no obvious signs of toxicity. (TIFF) [file pone.0092853.s005.tiff]

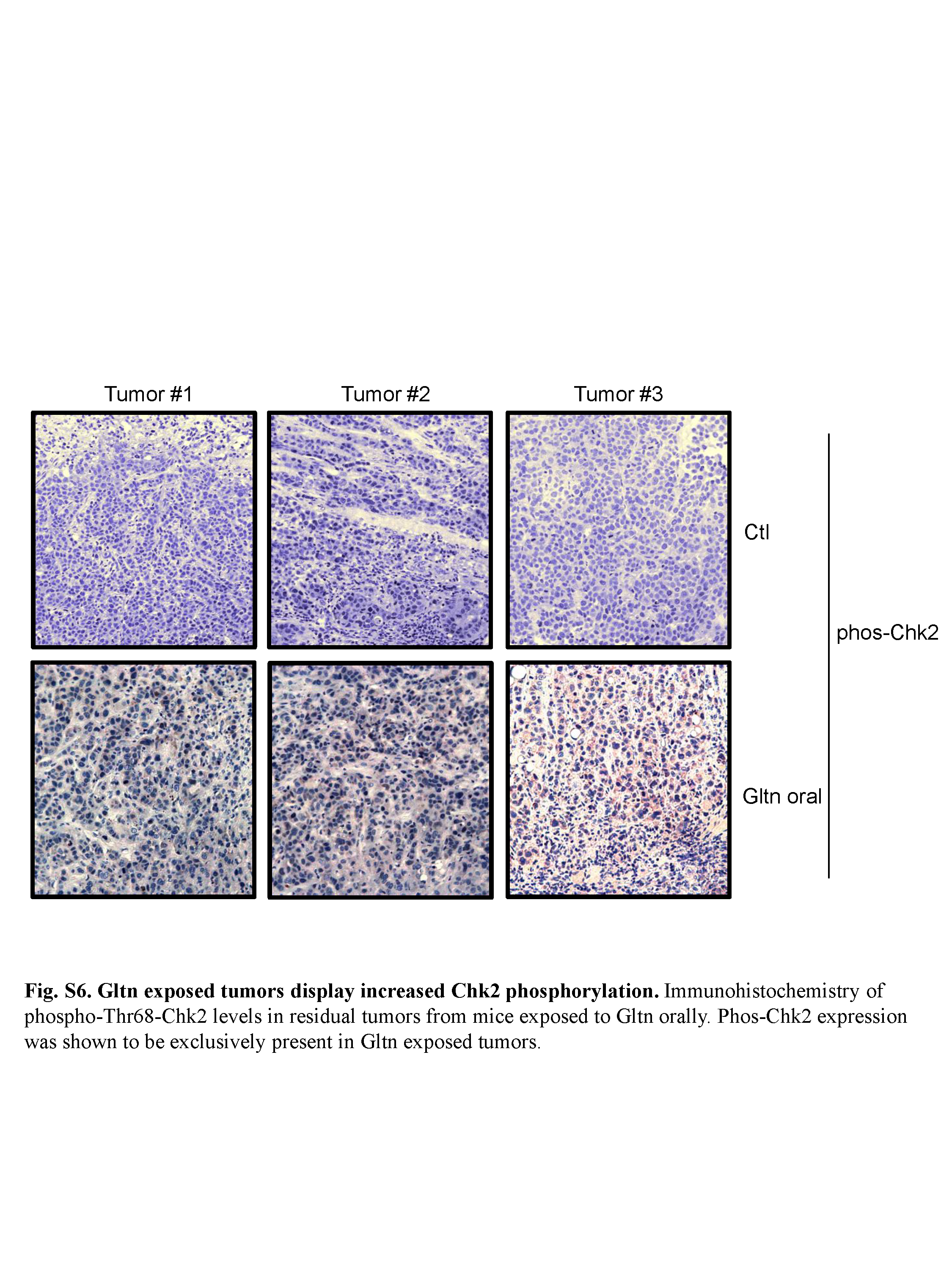

Supplement: Figure S6 — Gltn exposed tumors display increased Chk2 phosphorylation. (TIFF) [file pone.0092853.s006.tiff]

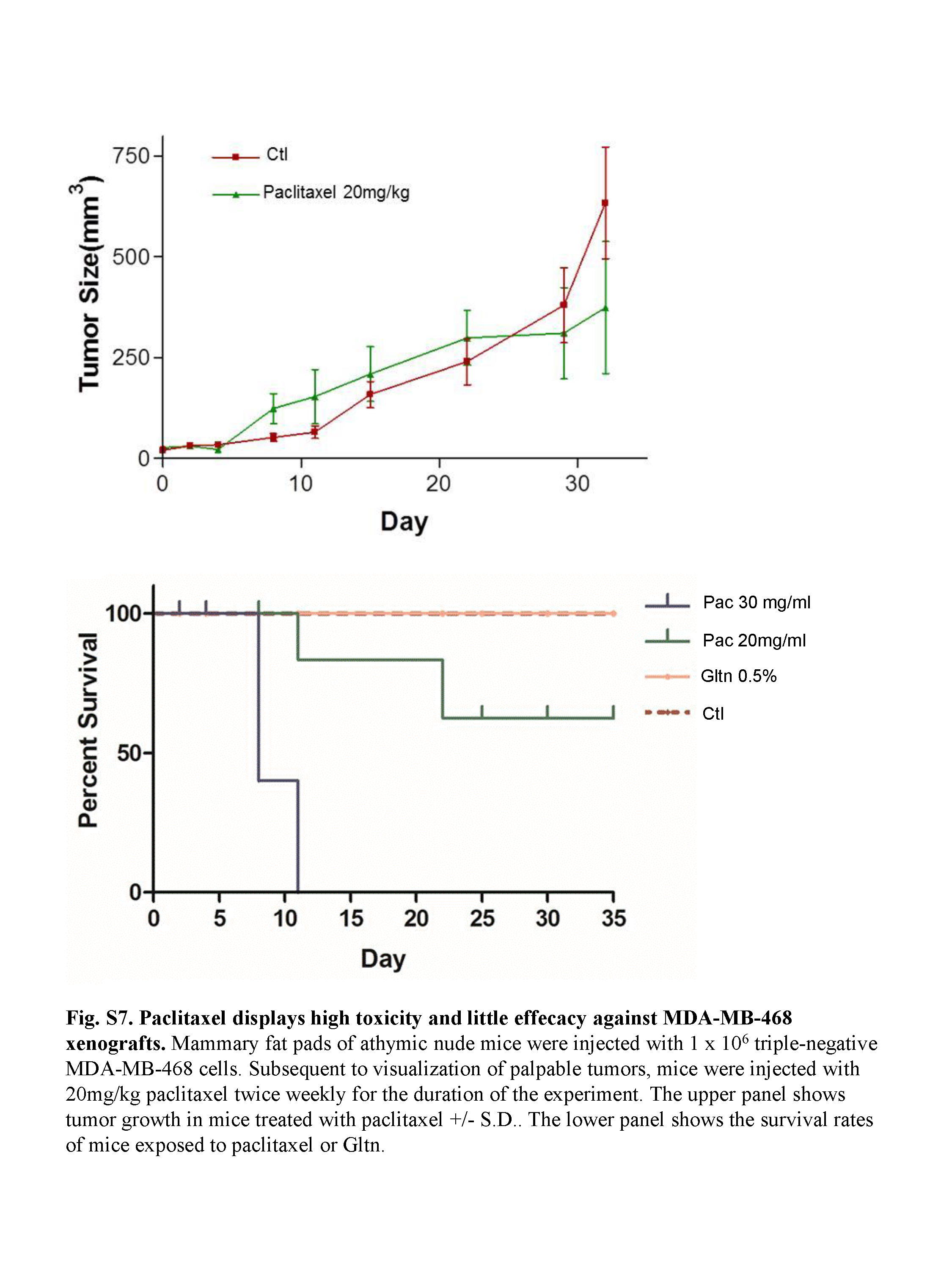

Supplement: Figure S7 — Paclitaxel displays high toxicity and little effecacy against MDA-MB-468 xenografts. (TIFF) [file pone.0092853.s007.tiff]
